# Coffee as a Remedy for Whooping-Cough—Guyot

**Published:** 1861-08

**Authors:** 


Coffee as a Remedy for Whooping Cough.—In Dr. George D. Gibbs' work on whooping cough, published in London in 1854, he quotes Dr. Jules Guyot as authority for recommending strong coffee as a specific for the cure of this disease. We [*Boston Med. and Sur. Jour.*] have tried it in several instances with marked effect. In one case, the patient being a little girl six years of age, there was not a single "whoop" after she began to take it. She took a tablespoonful and a half of very strong coffee, sweetened, but without milk, three times daily. A younger child, in the same family, was well of the disease in three weeks; no other remedy was used in either case. In another instance, in which we have recently tried it, the same happy result followed, the "whooping" symptom being at once arrested, and the complaint coming to a speedy termination. It is difficult to fix the dose definitely, and this may account for the unsatisfactory result in one or two instances we have heard of, in which a small dose was given. Another important consideration which should not be lost sight of, is, that three-quarters, probably, of what is drank for coffee, is made from nothing but peas or beans. The only sure method is to get the coffee berry itself, and have it burnt under one's own eye. The decoction should then be given as strong as possible, and in a quantity only short of enough to cause the unpleasantly stimulating effects of this beverage. Children take it very readily. The last patient referred to above, was only eighteen months old, and took, once a day, half a cup of coffee thus prepared, without the least noticeable injurious effect. As whooping cough is rather prevalent at the present time, we have thought it worth while to remind our readers of so simple and pleasant a remedy for it.

DOLBEAN reports two cases of prolapsed bowel, (one in a girl three years old, the other in a boy five years old,) both of which were cured by the subcutaneous injection of sulphate of strychnia, 30 centigrammes to 30 grammes of water. The canula was introduced one centimetre to the right of the anus to the depth of 0.5 centimetre, and 10-11 drops of the solution injected.

